# Differential transcriptional and epigenetic signatures in CD4^+^ T cells separate enthesitis related and psoriatic juvenile idiopathic arthritis – a pilot study

**DOI:** 10.1016/j.jtauto.2026.100397

**Published:** 2026-08-29

**Authors:** O. McClurg, J. Hawkes, F. Bates, A.S. Carvalho, C.E. Pain, L.J. McCann, C.M. Hedrich

**Affiliations:** a Department of Women's and Children'd Health, Institute of Life Course and Medical Sciences, University of Liverpool, United Kingdom; b Alder Hey NIHR Clinical Research Facility, United Kingdom; c Department of Rheumatology, Alder Hey Children's NHS Foundation Trust, United Kingdom

**Keywords:** Inflammatory arthritis, JIA, Psoriatic, Enthesitis, ERA, Methylation, Transcription

## Abstract

Juvenile idiopathic arthritis (JIA) is the most common musculoskeletal rheumatic disease affecting children. Following the currently used International League of Associations for Rheumatology (ILAR) classification, JIA encompasses seven clinically defined subtypes all of which share chronic inflammatory arthritis as a unifying symptom. Re-classification of JIA based on molecular signatures has been proposed and promises future patient stratification based on disease mechanisms and target-directed therapeutic interventions. This study applied flow cytometric immune cell profiling alongside mRNA expression analysis (NanoString inflammation panel) and DNA methylation profiling (Illumina EPIC450K arrays) to identify molecular signatures associated with enthesitis-related arthritis (ERA) or juvenile psoriatic arthritis (jPsA) subtypes of JIA. Disctinct gene expression and DNA methylation signatures in peripheral blood CD4^+^ T-cells uniquely associate with ERA versus jPsA. Disease-associated sigantures associate with immune memory, immune checkpoint disturbances, and inflammatory signaling. These exploratory findings provide preliminary evidence for molecular differences between ERA- and jPsA and support further evaluation of genetic and/or epigenetic stratification approaches in larger cohorts, to deliver towards individualised and target-directed treatments.

## 1 Introduction

Juvenile idiopathic arthritis (JIA) is the most common chronic rheumatic disease in children, and encompasses a clinically heterogenous group [1]. In the absence of a widely accepted molecular classification system, JIA subtypes are defined following International League of Associations for Rheumatology (ILAR) criteria [2]. Whilst grouped under a shared diagnostic umbrella, and sharing a ‘chronic inflammatory arthritis’ as a key feature, individual JIA subtypes may differ in clinical presentation, genetic associations and treatment response [3]. However, overlapping clinical appearance may result in misclassification and delayed introduction of ‘subtype-specific’ treatment [4].

Among the clinically overlapping JIA subtypes most prone to misclassification are enthesitis-related arthritis (ERA) and juvenile psoriatic arthritis (jPsA). While in adults psoriatis-associated inflammatory arthritis in most patients presents years after the onset of skin disease, and estimated 50% of children with psoriatic JIA experience arthritis before they develop skin disease [5,6]. The presence of enthesitis in a subset of patients with jPsA may further complicate separation from ERA. Diagnostic delay can result in the development of enthesitis in up to 60% of late-onset jPsA patients [6,7]. Indeed, in adult rheumatology, counterparts are considered part of the axial spondylarthritis/psoriatic arthritis spectrum [8]. Thus, better identification of shared and distinct immune pathways associated with these forms of JIA may allow molecular patient stratification towards new target-directed treatments [9,10].

Currently, little is known about the exact cellular and molecular pathophysiology of JIA. In jPsA and ERA, CD4^+^ T-cells have been suggested to play a central role in immune activation, associated pathological cyotkine release and tissue inflammation [9,10]. Important steps on the way to ‘loss of immune tolerance’ in JIA include altered antigen presentation, changes to T-cell differentiation and regulatory checkpoint function [11]. However, peripheral immunophenotyping has not yet delivered a complete understanding of altered immune function and pathomechanisms in jPsA and ERA that may be used clinically.

This study investigated transcriptional profiles in CD4^+^ T-cells from patients with jPsA and ERA with DNA methylation patterns, an epigenetic mechanism centrally invovled in tissue differentiation and immune function [12,13]. Altered DNA methylation and associated transcriptional signatures have, indeed, been linked with several T-cell driven autoimmune/inflammatory diseases across age groups, including psoriasis [14]. Previous studies in JIA focused on DNA methylation alone, not considering associated gene expressions, and delievered mixed results that may reflect the complex disease heterogeneity, and mixed cell populations [15]. Limited JIA subtype analyses, mainly focusing on oligo and polyarticular JIA populations, may have challenged data interpretation. Integrated analyses of gene expression alongside DNA methylation in purified defined CD4^+^ T-cell subsets from clinically defined patient populations may provide a more informative view of disease-associated immunoregulatory mechanisms Thus, we investigated ‘non-regulatory’ CD4^+^ T-cells from healthy participants, ERA patients and jPsA patients.

## 2 Methods

### 2.1 Sample collection

Between 2020 and 2022 healthy participants/controls (HC; N = 5), enthesitis-related arthritis (ERA; N = 5) and psoriatic arthritis patients (jPsA; N = 5) were enrolled in the study at the Department of Rheumatology, Alder Hey Children's NHS Foundation Trust, Liverpool, UK. The study was delievered adhering to the declaration of Helsinki, and all patients and/or their legal guardians provided written informed consent (Integrated Research Application System (IRAS): 242867). Patients were classified following the ILAR classification criteria for JIA [2]. Juvenile Arthritis Disease Activity Scores (JADAS) were calculated at recruitment to approximate disease activity [16]. Whole blood samples were collected from both JIA patients and HC, and peripheral blood mononuclear cells (PBMCs) were isolated using Histopaque (Sigma, Gillingham, UK) density centrifugation, following incubation with HetaSep (Stemcell, Cambridge, UK).

### 2.2 Flow cytometry and cell sorting

Fluorescence-activated cell sorting (FACS) was applied to phenotype PBMCs and isolate CD4^+^ T-cells excluding regulatory T-cells (Tregs) (defined as CD3^+^CD4^+^CD25^+^CD127^low^) using the following antibodies: Pacific Blue anti-CD4 (OKT4); Fluorescein Isothiocyanate (FITC) anti-CD3 (OKT3); Phycoerythrin (PE) anti-CCR7 (G043H7); Allophycocyanin (APC) anti-CD45RA (HI100), Allophycocyanin-Cyanine 7 (APCCy7) anti-CD8 (SK1), Phycoerythrin (PE) CF594 anti-CD25, Phycoerythrin-Cyanine5 (PE-Cy5) anti-CD127 and Peridinin chlorophyll protein-Cyanine5.5 (PerCP-Cy5.5) anti-CD279 (PD1). ‘Non-regulatory’ CD3^+^CD4^+^CD25^−^ T-cells were selected to investigate alterations to ‘effector’ CD4^+^ T-cell phenotypes with minimal dilution through background signal [17]. Flow cytometry results were analysed using FlowJo™ (v10) Software. Figures were obtained with GraphPad Prism software (version 10.0); groups were compared using one-way analysis of variance (ANOVA) followed by Tukey's post hoc tests.

### 2.3 Gene expression and DNA methylation analyses

Genomic DNA and RNA were extracted from isolated CD3^+^CD4^+^CD25^−^ T-cells (All prep Kit, Qiagen) and stored at −150 °C for analysis. Gene expression was quantified with the Nanostring (Bruker, Massachusetts, USA) nCounter autoimmune panel using 100 ng of RNA. Data underwent quality control using the ‘*NACHO’* R package [18] followed by normalization and analysed using R (v 4.5.2) and the *‘nanostringr’* package [19]. Genes were defined as differentially expressed when having a p-value < 0.05, and a |Log2 fold change| > 1.5.

DNA methylation analysis was performed using DNA isolated from the same CD3^+^CD4^+^CD25^−^ T-cells using Illumina MethylationEPIC 450k arrays (Diagenode, Vienna, AT). DNA methylation profiles were analysed using R packages ‘*minfi’* [20] and ‘*ChAMP’* [21]*.* DNA methylation analysis was restricted to CpG probes associated with the differentially expressed genes identified using the NanoString nCounter autoimmune arrays. Quantile and Beta Mixture Quantile (BMIQ) normalization strategies were applied to raw data. Poor quality probes (p-value > 0.01) were identified and excluded as described previously [17]. Potential batch effects and technical covariates were assessed using ChAMP SVD analysis and identified batch effects were adjusted using ComBat empirical Bayes correction, as implemented in ‘*ChAMP’* [22]. Probe expression was defined as statically different between healthy controls, ERA- and jPsA groups applying a false discovery rate (FDR) < 0.05 and a delta beta value (|Δβ|) > 0.05.

### 2.4 Biological pathway annotation

*Kyoto Encyclopaedia of Genes and Genomes* (KEGG) [23] pathway analysis and *Gene Ontology* (GO) [24,25] term analysis for biological processes functional enrichment were performed using the *‘EnrichR’* packages in R [26]. Pathways were considered enriched if they had an adjusted p-value < 0.05 following correction for multiple hypotheses using the Benjamini-Hochberg method.

## 3 Results

### 3.1 Demographics

Fifteen age- and sex-matched participants of European ancestry were recruited to this study, including 5 healthy participants/controls (HC), 5 patients with ERA and 5 patients with jPsA. No differences were observed in relation to JADAS scores between the JIA groups, nor were there differences in patients receiving methotrexate or biological disease modifying antirheumatic drugs (DMARDs) (Table 1).

**Table 1:** Participant demographics.

	HC	ERA	jPsA	*p-value*
**Total**	5	5	5	NS
**Male (%)**	3 (60)	4 (80)	3 (60)	NS
**Age, median (IQR), years**	11 ± 5	14 ± 3	8 ± 6	NS
**JADAS score, median (IQR)**	N/A	7 ± 11.2	2 ± 10	NS
**Patients receiving bDMARDs, n (%)**	N/A	2 (40)	2 (40)	NS
**Patients receiving MTX, n (%)**	N/A	3 (60)	4 (80)	NS

Table of the clinical demographic data from the healthy control (HC) and JIA cohorts. IQR – Interquartile range. JADAS – Juvenile Arthritis Disease Activity Score composed of Joint Count, Physician and patients/parents global assessment and erthrocyte sedimentation rate. bDMARDs – biological disease modifying arthritis drugs, MTX – Methotrexate. Data presented as % of cohort unless stated otherwise.

### 3.2 Immunophentyping

**Fig. 1:**
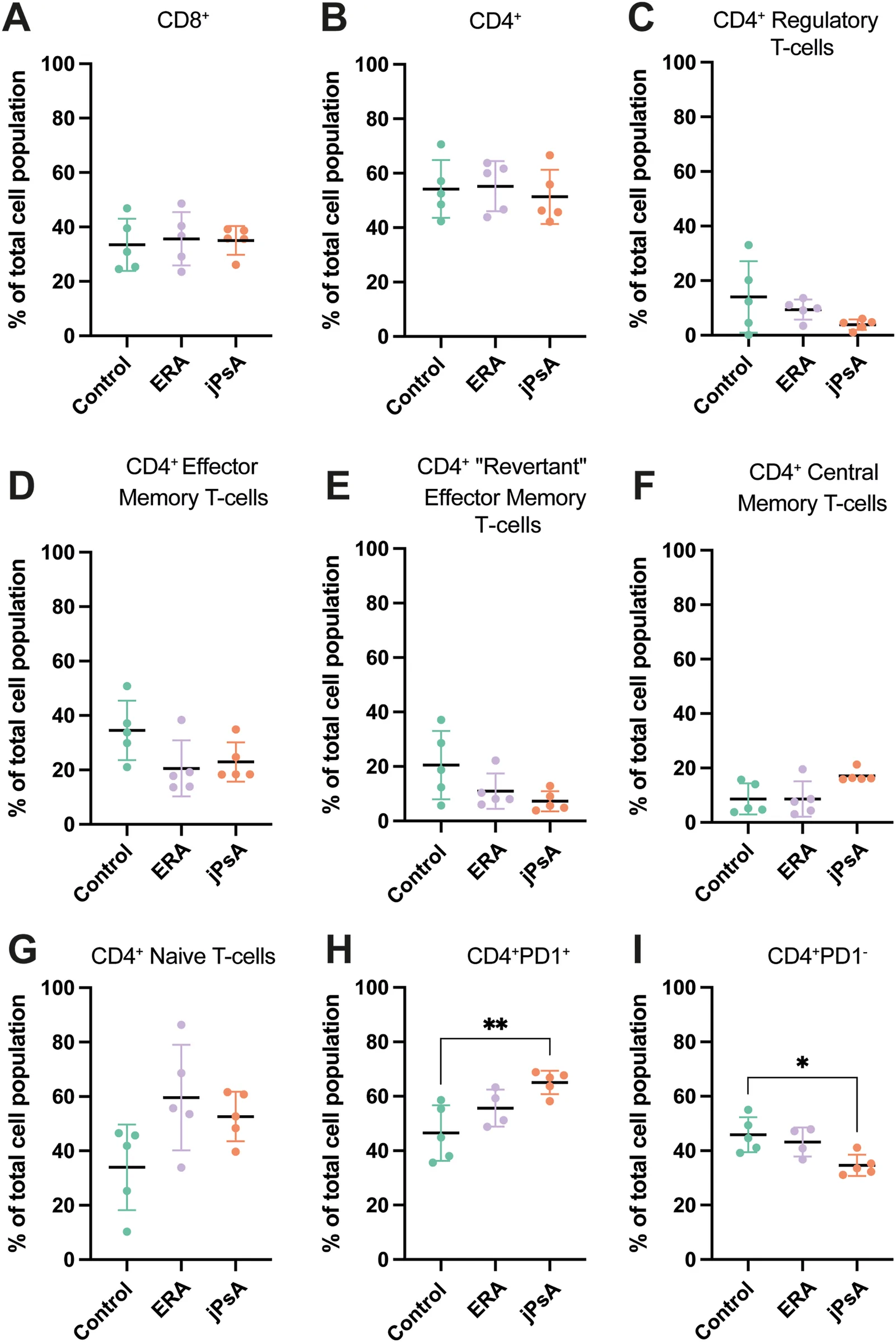
**Immune phenotyping of PBMCs isolated from the blood of child healthy controls, ERA-JIA or jPsA patients.** (A) CD3^+^CD8^+^ T cells, (B) CD3^+^CD4^+^ T cells, (C) Regulatory T cells (CD3^+^CD4^+^CD25^+^CD127^low^), (D) Effector Memory T cells (CD3^+^CD4^+^CCR7^−^CD45RA^−^), (E) “Revertant” Effector Memory T cells (CD3^+^CD4^+^CCR7^−^CD45RA^+^), (F) Central Memory T cells(CD3^+^CD4^+^CCR7^+^CD45RA^−^), (G) Naïve T cells (CD3^+^CD4^+^CCR7^+^CD45RA^+^), (H) CD4^+^PD1^+^ T cells, and (I) CD4^+^PD1^-^ T cells. Data analysed using one-way Anova and corrected for multiple comparisons using Tukey's test. Gating was performed using FlowJo 10.10.0. Control: Healthy participants; ERA: enthesitis-related arthritis; jPsA: juvenile psoriatic arthritis.

Proportions of CD8^+^ T-cells, total CD4^+^ T-cells, regulatory CD4^+^ T-cells (CD3^+^CD4^+^CD25^+^CD127^low^), effector memory CD4^+^ T-cells (CD3^+^CD4^+^CCR7^−^CD45RA^−^) and ‘revertant’ effectory memory CD4^+^ T-cells (CD3^+^CD4^+^CCR7^−^CD45RA^+^) were consistent between groups (Fig. 1A–D).

Moderate increases were identified in central memory CD4^+^ T-cells (CD3^+^CD4^+^CCR7^+^CD45^−^) (Fig. 1F) among jPsA patients when compared to HC (p = 0.05) and ERA patients (p = 0.05), alongside elevated (p = 0.05) CD4^+^ naïve T-cells (CD3^+^CD4^+^CCR7^+^CD45^+^) in ERA patients (Fig. 1G) as compared to HC or jPsA patients. We observed increased expression of the regulatory programmed death (PD)-1 surface protein on CD4^+^ T-cells from jPsA patients (Fig. 1H and I) when compared to controls.

### 3.3 Gene expression profiling

**Fig. 2:**
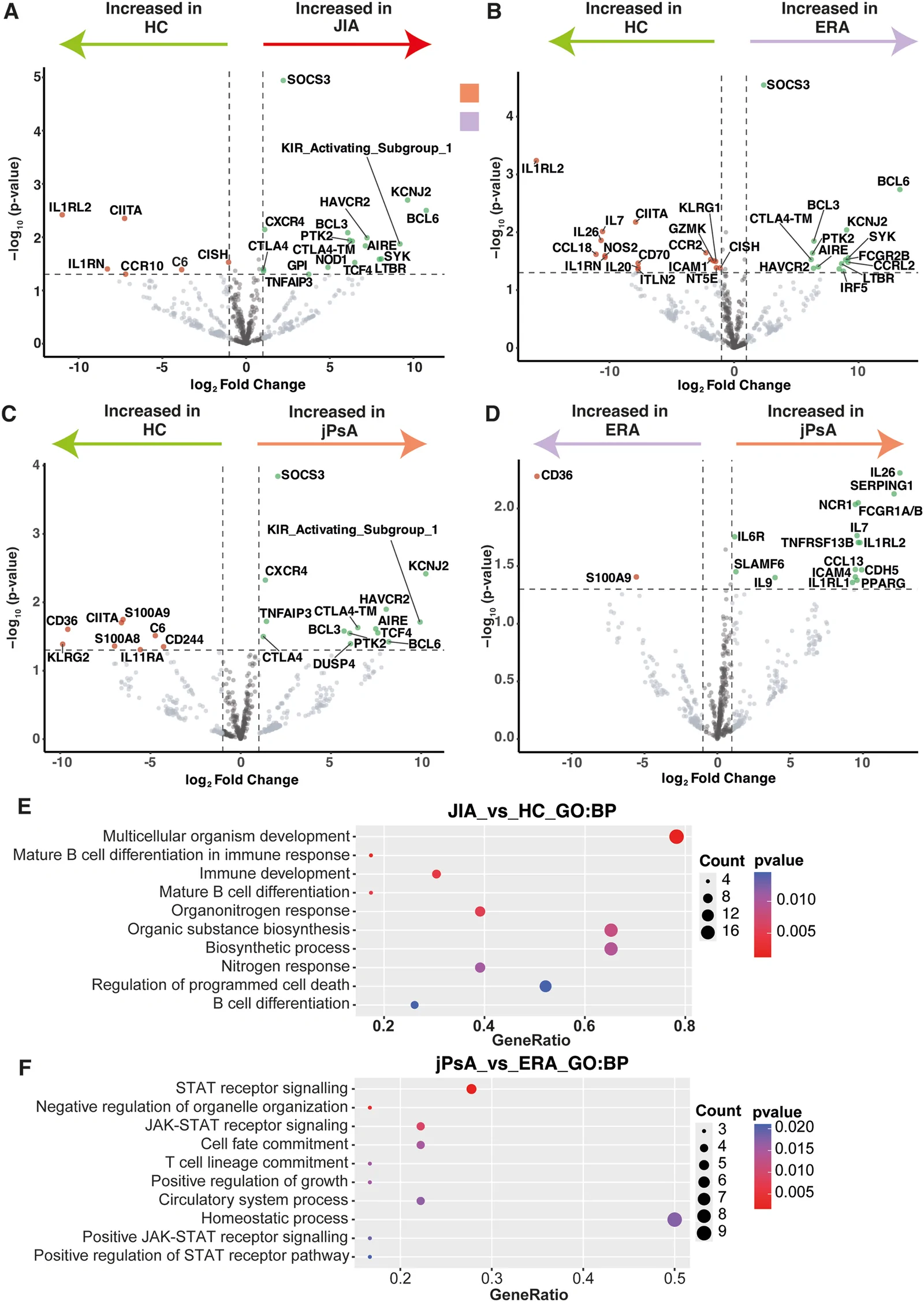
**Gene expression profiling of HC and JIA CD4^+^ ‘non-regulatory’ T-cells using nanostring autoimmune arrays.** RNA was isolated from CD4^+^ nonregulatory T-cells and quantified using qubit. 50 ng of RNA was run on nanostring autoimmune arrays. Gene expression was quantified as the ratio between the gene counts of target genes and the geometric mean of housekeeping gene counts. DEGs are identified as having a p-value < 0.05 and a |Log2 FC| > 1.5. (A) Volcano plot of genes differentially expressed between all JIA patients and healthy controls. (B) Volcano plot of genes differentially expressed between ERA-JIA and HC patients. (C) Volcano plot of genes differentially expressed between jPsA and HC patients. (D) Volcano plot of genes differentially expressed between jPsA and ERA-JIA patients. Data analysed in R (v 4.3.2) using packages ‘nanostringr’ and visualised using ‘EnhancedVolcanoPlot’. HC: Healthy participants; JIA: Juvenile idiopathic arthritis, ERA: enthesitis-related arthritis; jPsA: juvenile psoriatic arthritis.

**Fig. 3:**
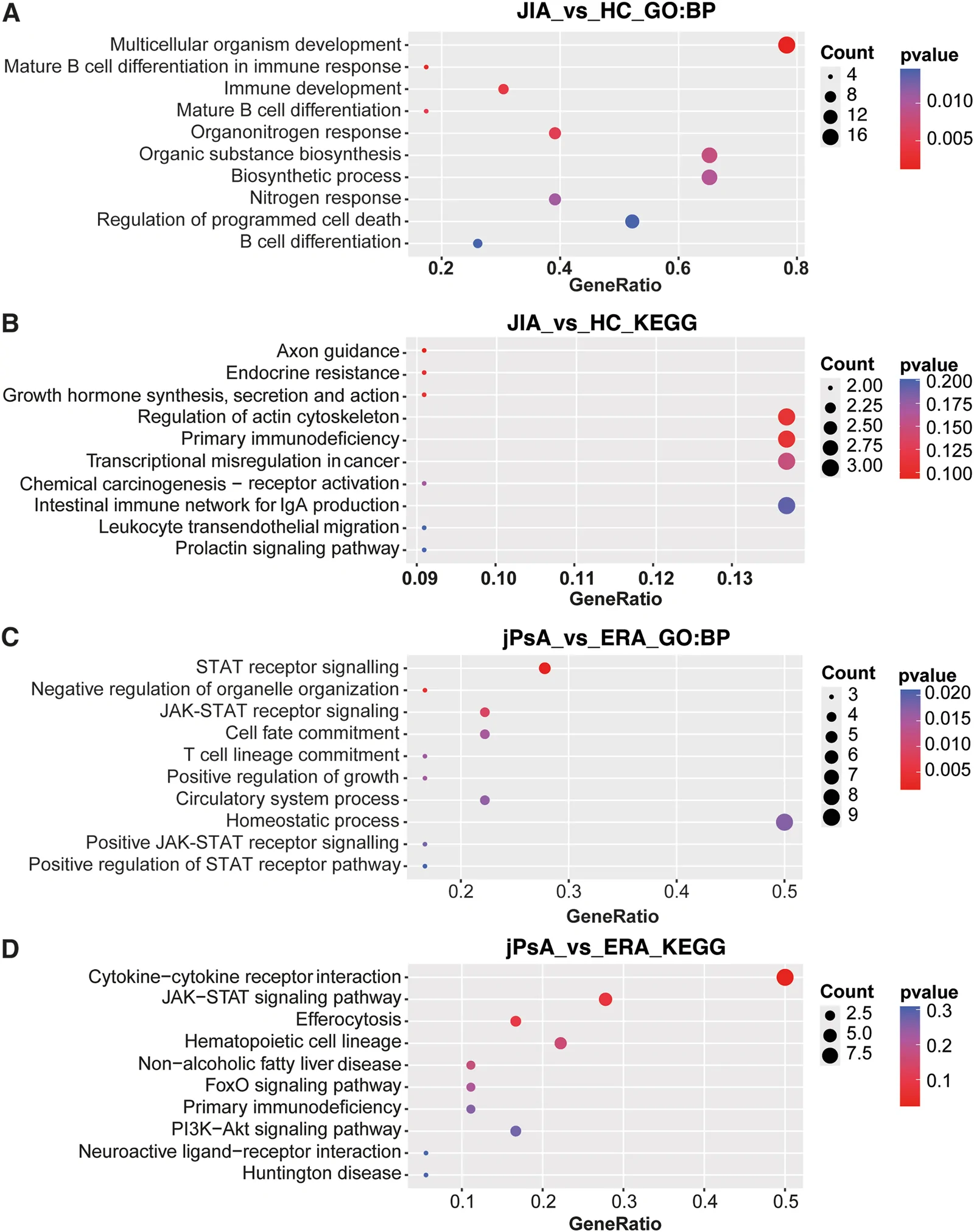
**Over-enrichment analysis of differentially expressed genes between JIA and healthy controls, and jPsA and ERA-JIA patients.** Genes identified to be differentially expressed (p-value < 0.05, |Log2 FC| > 1.5) using Nanostring autoimmunity arrays underwent functional enrichment analysis in R (v4.3.2) using the ‘EnrichR’ package. (A) Enriched GO:BP terms of genes differentially expressed between ‘all’ JIA patients and healthy controls. (B) Enriched KEGG terms from genes differentially expressed between ‘all’ JIA patients and healthy controls. (C) Enriched GO:BP terms of genes differentially expressed between jPsA and ERA-JIA patients. (D) Enriched KEGG terms from genes differentially expressed between psJIA and ERA-JIA patients. HC: Healthy participants; JIA: Juvenile idiopathic arthritis, ERA: enthesitis-related arthritis; jPsA: juvenile psoriatic arthritis.

Because CD4^+^ T-cell dysfunction has been linked with the pathophysiology of JIA [27,28] we investigated mRNA expression in ‘non-regulatory’ CD4^+^ T-cells (Auto-immune panel, NanoString). Of 579 genes profiled, 26 were differentially expressed between all ‘JIA’ (ERA + jPsA patients, n = 10) and HC patients (Fig. 2A), 23 displayed a LogFC > 1.5 change (e.g. Increased; *SOCS3, KCNJ2, BLC6,* Decreased*; IL1RN, IL1RL2, CIITA)*. Functional enrichment analysis (GO:BP and KEGG) identified enrichment for pathways associated with immune developmental (e.g. Immune development (GO:BP), primary immunodeficiency (KEGG)), cell apoptosis (Regulation of programmed cell death (GO:BP)), and migration (Axon guidance, Regulation of actin cytoskeleton, Leukocyte transendothelial migration (KEGG)) (Fig. 3A and B).

Comparing ERA patients to healthy controls, we observed 33 genes dysregulated, of which 29 exhibited LogFC > 1.5 change (Fig. 2B). ERA patients showed altered expression of inflammatory mediators (*IL6R, IL7/7R, CCRL2, CCR2, CCL18*), immune checkpoint-associated/negative immune regulator genes (*CTLA4-TM, HAVCR2, FCGR2B*) and JAK-STAT signalling (*SOCS3, CISH)* (Fig. 2B) when compared to healthy controls. Correspondingly, these genes were enriched for pathways involved in JAK-STAT FcyR signalling, cytotoxicity and cytokine signalling (Supplementary Fig. 1A and 1B).

jPsA patients exhibited dysregulation of 22 genes when compared to healthy controls (Fig. 2C) with a LogFC > 1.5. Genes that were regulated were associated with innate inflammatory markers (*S100A8,* S100A9*, BCL3, TNFAIP3),* negative immune regulation (*SOCS3, DSUP4, CTLA4)* and T-cell activation (*BCL6, TCF4, CIITA).* Functional enrichment analysis identified enrichment for GO:BP terms involved in wound healing, cell growth and cell-matrix adhesion (Supplementary Fig. 1C) and KEGG terms for efferocytosis and actin cytoskeletal regulation (Supplementary Fig. 1D).

Comparing jPsA to ERA, markedly fewer genes are changed. 17 genes were differentially expressed with a LogFC>1.5 (Fig. 2D). We observed increased expression in jPsA patients for genes associated with Th17 barrier-type inflammation, such as *IL26, IL6R*, *IL1RL1*, *IL1RL2* and S100A9 alongside regulated expression of genes involved in chemotaxis and cell trafficking, such as increased *CCL13*, *S1PR1*, *CDH5*, *ICAM4* and *IL6R*. Correspondingly, GO:BP analysis identified enrichment for terms involved in JAK-STAT signalling and T-cell lineage commitment (Fig. 3C) whereas KEGG analysis identified fewer significant terms but still identified enrichment for cytokine-cyotkine receptor interactions (Fig. 3D), likely reflecting the regulated expression of *IL6R*, *IL1RL1*, *IL1RL2* and *IL7R*.

### 3.4 DNA methylation

**Fig. 4:**
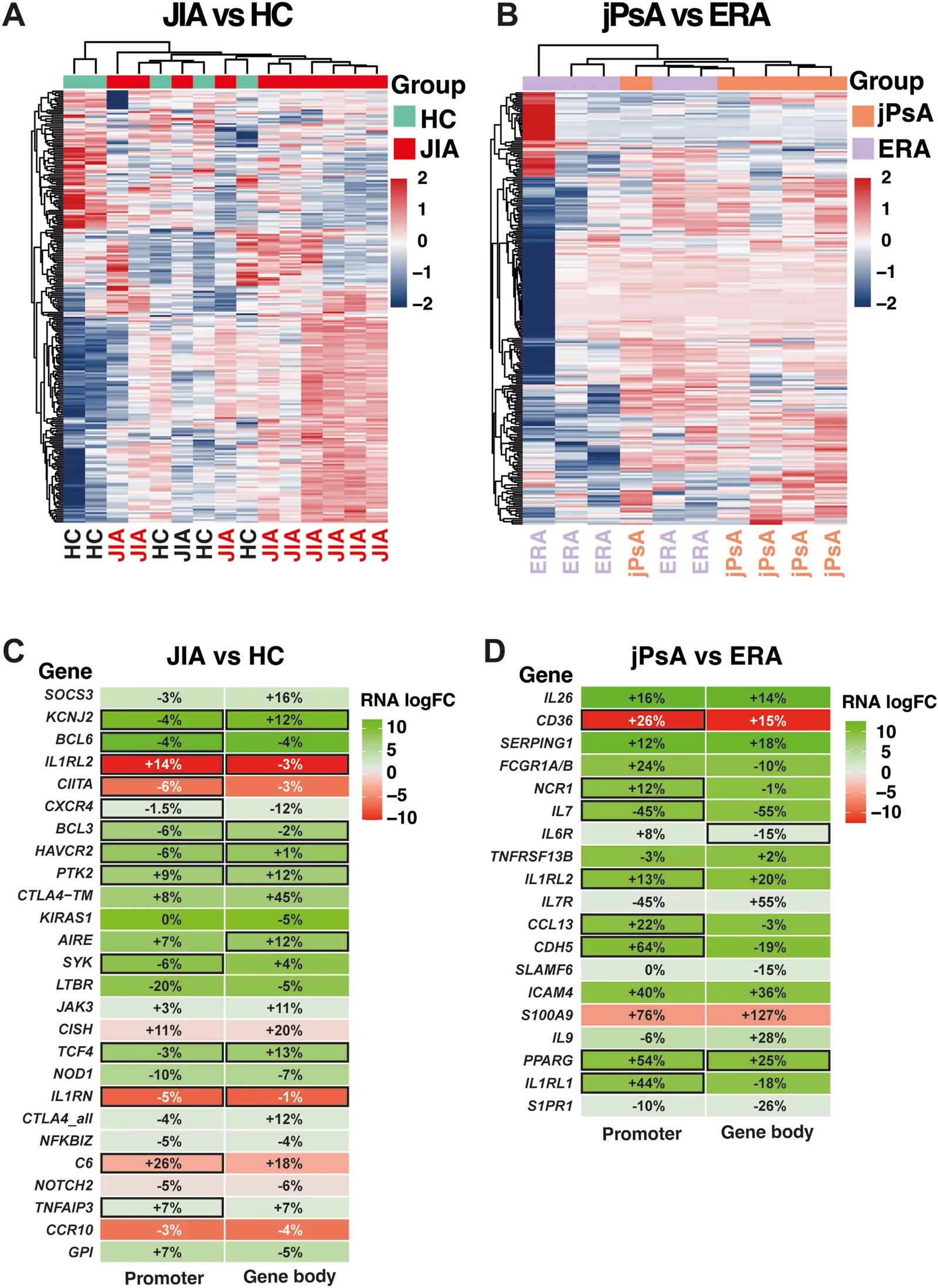
**Differential methylation of differentially expressed genes between JIA subgroups and healthy controls.** Differentially expressed genes identified using Nanostring arrays had their DNA methylation quantified using EPIC 450k arrays. (A) Hierachical clustering of the top 250 probes between HCs and JIA patients (FDR < 0.05, |Δβ| > 0.05). (B) Hierarchical clustering of the top 250 probes between ERA-JIA and jPsA patients (FDR < 0.05, |Δβ| > 0.05). (C) Heatmap of differential gene expression and DNA methylation between JIA and HC patients. (D) Heatmap of differential gene expression and DNA methylation between jPsA and ERA-JIA patients. Green indicates increased gene expression; red indicates reduced gene expression. Grey indicates no significant change in expression for that comparison. Numbers overlaid represent % change of DNA methylation for CpGs significantly associated with promoter regions or gene body regions. Black outlines indicate genes that were both significantly differentially expressed and had significant changes in methylation (p = 0.05). Genes with >1 CpG probe for either the promoter or gene body were transformed into an average. Promoter regions were defined as TSS200 and TSS1500. HC: Healthy participants; JIA: Juvenile idiopathic arthritis, ERA: enthesitis-related arthritis; jPsA: juvenile psoriatic arthritis.

Next, DNA methylation was investigated across the 62 genes that showed differential mRNA expression across the 4 contrasts (JIA vs healthy, ERA vs healthy, jPsA vs healthy and jPsA vs ERA (Fig. 2). Using hierachical clustering of the top 250 differentially methylated probes, we first investigated how all JIA patients compared to HC patients. JIA patients clustered moderately by disease, with 6 JIA patients cleanly distinct from HC, and 4 patients showing CpG methylation correlating with HC patients (Fig. 3A). When comparing jPsA patients to ERA patients, one jPsA patient was misclassified with ERA patients (Fig. 3C). When comparing ERA to HC, one ERA patient was misclassified with HC patients, and when comparing jPsA to HC one HC patient was misclassified with the jPsA patients (Supplementary Fig. 2). Finally, for those genes that were differentially expressed between JIA and HC patients, and jPsA vs ERA patients, we looked at how gene-associated probe methylation was altered in both the promoter and gene body regions (Fig. 4C and D)

Out of the 23 genes differentially expressed between JIA and HC patients (Figs. 2A), 13 genes showed differential DNA methylation. Eleven of these genes had significantly differential promoter methylation, compared to 8/13 genes showing significantly differential methylation of their gene bodies (Fig. 4C, black boxes).

Of the 29 differentially expressed genes between ERA patients and HC (Fig. 2B), we observed differential methylation in 19 genes. Of these 19 differentially methylated genes, 11 had differentially methylated promoter regions, and 11 had differentially methylated gene bodies. *BCL3, LTBR, PTK2* and *SYK* had both their promoter and gene body regions differentially methylated.

Of the 22 regulated genes between jPsA patients and HC (Fig. 2C), jPsA had 17 differentially methylated genes. 15 of these were differentially methylated in their promoter regions, comapred to 12 differentially methylated in their gene body regions. *C6, CIITA, DUSP4, HAVCR2, IL1*1RA*, KLRG2, PTK2, S100A9,* and *TCF4* were differentially methylated in both their promoter and gene body regions.

Finally, we investigated how promoter and gene body methylation differed between jPsA and ERA patients. Of the 17 genes differentially expressed (Fig. 2D), 9 genes were differentially methylated. 7 of these were differentially methylated at their promoter regions and 3 were differentially methylated in their gene body regions (Fig. 4D). Only *PPARG* was differentially methylated at both its promoter and gene body.

## 4 Discussion

Published evidence of genetic and epigenetic regulation from peripheral blood-derived CD4^+^ T-cells is scarce, with many studies failing to identify consistent differences [15,29]. This is further confounded by the impacts of disease modifying antirheumatic drugs (DMARDs), such as methotrexate [[30], [31], [32]]. Consequently, studies focus on the more common JIA subtypes, such as oligoarticular JIA and polyarticular JIA, resulting in less common subtypes, such as psoriatic JIA and enthesitis-related arthritis, remaining significantly understudied. This exploratory pilot study, to the best of our knowledge, for the first time integrated transcriptional signatures and DNA methylation patterns of CD4^+^ T-cells from patients with ERA, jPsA as compared to healthy patients.

Peripheral blood immune cell phenotyping revealed a moderate increase in central memory CD4^+^ T-cells (CD3^+^CD4^+^CCR7^+^CD45^−^) in jPsA patients when compared to patients with ERA or healthy participants (p = 0.05). This may reflect a chronic inflammatory profile in jPsA and agrees with previous reports on altered central memory CD4^+^ T-cells (defined as CD4^+^CCR7^+^ and CD4^+^CCR7^+^CXCR5^+^) [33], or perturbations of TH17/Th22-associated clusters in adult psoriasis patients [34].

Moreover, elevated naïve CD4^+^ T-cell proportions in ERA patients (and to a lesser non-significant extent in jPsA patients) as compared to healthy participants, may reflect an increased immune cell turnover and mobilisation of naïve T-cells from the bone marrow [35]. However, in systemic lupus erthymatosus (SLE), recent reports highlighted the presence of epigenetically reprogrammed ‘naïve’ CD4^+^ T-cells coexisting alongisde ‘physiological’ naïve CD4^+^ T-cells. Reprogrammed ‘naïve’ T-cells differentiate into Th2 or Th17 cells significantly quicker than ‘healthy’ naïve T-cells, due to demethylation of the promoters for genes such as *IFI44L, MX1*, and *STAT1* [36]. Indeed, analysis of genes differentially expressed between patients with ERA and HCs associated with Th2 and Th17 cell lineage differentiation/function, including JAK-STAT signalling, cytotoxicity and cytokine signalling, likely driven by *CTLA4*, *BCL6*, *BCL3*, *CISH* and *SOCS3* expression. The seeming discordance between the ‘resting’/naïve CD4^+^ T-cell phenotype alongside the here identified ‘active’ transcriptional signature underscores limitations of relying solely on the expression of cell surface markers to assess immune competence.

In CD4^+^ T-cells from jPsA patients gene expression signatures linked more closely to chronic inflammation (*TNFAIP3* [37]*, CTLA4* [38]*, HAVCR2* [39]) and wound healing (*S100A8/A9* [40]*, IL1*1RA [41]*, CXCR4* [42]). This is consistent with previous reports on psoriasis-associated expression of genes involved in tissue remodelling and barrier-associated inflammation [43,44]. Morever, we observed an increased proportion of CD4^+^PD1^+^ T-cells in jPsA patients when compared to HC and patients with ERA. The PD-1 surface co-receptor plays a key role in effector T-cell regulation and immune exhaustion [45]. Increased PD-1 positivity has previously been observed in psoriatic skin plaques compared to healthy controls [46] as well as CD8^−^CD4^−^ (double negative) T-cells in psoriasis [47]. Interestingly, PD-1 positivity increased in adult psoriatic CD4^+^ T-cells [48] compared to healthy controls, but not in psoriatic arthritis, indicating a role for the down-regulation of PD-1 in CD4^+^ T-cells in psoriatic arthritis in adults, but not juveniles. Increased expression of PD-1 has been discussed as a surrogate for immune exhaustion and chronic inflammation in psoriasis [49].

Alternatively, PD-1 expression may reflect a failed-brake mechanism. Under physiological conditions, T peripheral regulator (Tpr) cells (FoxP3^+^PD-1^int^) identify and suppress pathogenic T phollicular helper cells. In active PsA, synovial fluid contains high levels of soluble PD-1, a free floating version of the receptor shed from the cell surface, that intercepts PD-L1 and thus preventing T-cell exhaustion [50]. Whilst we did not detect differential expression of *PDCD1* at the mRNA level we observed increased expression of genes downstream of PD-1, such as dual-specificity protein phosphotase 4 (DUSP4). DUSP4 is involved in negative-regulation of MAPK signalling, a pathway that has been shown to be dysregulated in psoraisis [51] and may therefore represent a negative-regulatory mechanism linked to the increased CD4^+^PD1^+^ T-cell proportions.

Increased expression of *CTLA-4,* another ‘immune checkpoint’ in CD4^+^ T-cells from patients with ERA and jPsA when compared to HCs may reflect another attempt of the immune system to self regulate [52]. Alternatively, increased expression of *CTLA4* may be associated with previously reported numerical expansion of CD4^+^CD28^−^ T-cells, unresponsive to CTLA-4 and B7 inactivation. This may increase *CTLA-4* expression without functional effect [53]. Indeed, dysregulation of *CTLA-4* has been implicated in JIA, with polyarticular JIA patients showing increased soluble PD-1 and CTLA4 in the peripheral blood [54] further highlighted by the success of biological DMARDs activating *CTLA-4* in treated polyarticular JIA *CTLA-4* [55].

When comparing jPsA to ERA patients there were fewer differentially expressed genes, likely reflecting shared inflammatory activation between the JIA subtypes, however following over enrichment analysis we observed changes to pathways involved in cytokine signalling and T-cell lineage commitment reflected by increased *IL7, IL1R2, IL26, IL6R* and *IL9* expression in jPsA as compared to ERA patients. This likely represents changes to how CD4^+^ T-cells in these subtypes interperet cytokine-rich environments and commit towards functional states [56,57].

Dynamic DNA methylation patterns play a key role determining T-cell lineage differentiation [58]. Interrogation of the DNA methylation status of differentially expressed genes in CD4^+^ T-cells only allowed incomplete separation of ‘all’ JIA patients from HC, while more clearly discriminating jPsA from ERA patients. Notably, challenges with discriminating ‘all’ JIA patients from HCs may reflect overall ‘low’ disease activity in the study, as our JIA patients had relatively ‘low’ JADAS scores**.** Moreover, identifying differential DNA methylation signatures between healthy participants and JIA patients have been reported previously [28,59] and some authors suggesting 5-hydroxymethylation, an intermediate between DNA methylation and demethylation, to be more clearly associated with JIA [60].

When considering DNA methylation patterns in relation to gene expression, genes differentially expressed between groups, in several cases, also exhibited altered DNA methylation profiles affecting both their promoter region and gene-bodies. However, the relationship was not uniform in all cases. Whilst comparing samples from ‘all’ JIA patients to HC saw relatively coherent relationship between promoter methylation and changes to gene expression. Usually, reduced promoter methylation associated with increased gene expression and vice versa [58]. This was not the case when comparing samples from jPsA to those from ERA patients, where increased promoter methylation frequently associated with increased gene expression. However, increased methylation of gene bodies, such as *HACVR2,* has previously been associated with increased gene expression, an observation also seen in this study [52]. These observations may reflect previously activated T-cell biology, developmental history, treatment exposure or lineage priming, which is reflected in the functional enrichment analysis. Moreover, promoter annotations include CpGs at varying distances from the transcriptional start site, including regions that may correspond to alternative promoters, CpG shores or distal regulatory elements that were not captured in this analysis [61]. Thus, whilst data from this stuy suggests differential DNA methylation contributing to subtype-associated epigenetic immune programming, linking gene expression to promoter and gene body, DNA methylation should be approached cautiously.

Whilst this study delivers promising gene expression and DNA methylation signatures differentially associated with ERA versus jPsA, it is limited by the relatively small study populations in a rare disease area (n = 5 per group). It must also be noted that methotrexate can impact on DNA methylation profiles. However given the relatively fewer DMP's identifed when comparing HC, who do not receive methotrexate, to all JIA patients (7/10 patients received methotrexate) revealed fewer changes in gene expression and DNA methylation than expected, suggesting the impact of methotrexate on gene expression in this cohort to be low.

While this exploratory pilot offer molecular signatures that may support future patient stratification, it is limited by the relatively small cohort size (n = 5 per group). Thus, findings should be considered hypothesis-generating and require validation in larger independent studies. Treatment exposure varies between patients and may affect data interpretation. Notably, dozens to hundreds of genes are affected by exposure to methotrexate, but data relating clinical methotrexate response with altered molecular features are sparse. Comparing here identified DEGs to genes known to be affected by methotrexate reveal limited overlap [62]. Differential expression of the *SOCS3, TCF4* and *DUSP4* genes has previously been reported in PBMCs from JIA patients after 6 months of methotrexate exposure, and were upregulated in ERA (*SOCS3, TCF4)* or jPsA (*SOCS3, TCF4, DUSP4)* cohorts in this study [62]. Conversely, methotrexate-associated DEGs in T cells from rheumatoid arthritis patient [63] showed no overlap with DEGs identified here. Larger cohort studies including sub-cohorts of patients receiving defined treatment regimens are necessary to reliably assess the impact of methotrexate treatment on DEGs. Patients enrolled in this study had relatively ‘low’ disease activity (average JADAS scores <7). Published evidence suggests that, as disease activity reduces, transcriptional states may adopt a ‘less-than-active’ but ‘more-than-healthy’ inflammatory signature [64,65] while epigenetic changes may be more ‘stable’^67,68^, particularly in CD4^+^ T cells [64]. However, no studies are available investigatign this in the JIA subclasses presented in this paper, ERA and jPsA. Further studies, including samples from matched active/inactive JIA patients, larger cohorts and longitudinally collected samples are necessary to elucidate which molecular signatures associate with disease activity. Lastly, given the exploratory nature of this pilot study, differentially expressed candidate genes were identified using a nominal p-value <0.05 togeyher with an effect-size threshold of |log2FC| >1.5. This approach was used to retain potentially informative signals for subsequent integrative analysis using DNA methylation arrays where we applied FDR correction. This does not sufficiently control for gene-level false discovery and requires independent validation.

## 5 Conclusions

In conclusion, preliminary data from this study identified disctinct gene expression and DNA methylation signatures in peripheral blood CD4^+^ T-cells from patients with ERA versus jPsA. Disease-associated signatures underscore alterations to immune memory, immune checkpoint disturbances, and inflammatory signaling. These preliminary findings offer further evidence to the view that JIA subtypes diverge by molecular features more than by their (anyway overlapping) clinical presentation, promising molecular phenotyping-based re-classification of disease to allow individualised and target-directed treatments. Further validation in larger, independent cohorts will be required before such signatures can be considered for molecular patient stratification.

## CRediT authorship contribution statement

**O. McClurg:** Writing – review & editing, Writing – original draft, Visualization, Validation, Methodology, Investigation, Formal analysis, Data curation. **J. Hawkes:** Writing – review & editing, Resources, Data curation. **F. Bates:** Writing – review & editing, Resources, Project administration, Data curation. **A.S. Carvalho:** Writing – review & editing, Visualization, Methodology, Investigation, Data curation. **C.E. Pain:** Writing – review & editing, Resources, Project administration, Methodology, Data curation. **L.J. McCann:** Writing – review & editing, Resources, Methodology, Investigation, Data curation, Conceptualization. **C.M. Hedrich:** Writing – review & editing, Writing – original draft, Visualization, Validation, Supervision, Resources, Project administration, Methodology, Funding acquisition, Conceptualization.

## Ethics declaration

Written informed consent to take part in the study and to publish the article has been obtained from all participants or their legal representatives. The privacy rights of participants have been observed.

This study included organ or tissue donors. This study includes human biological material and consent was obtained by donors, or their next of kin or legal representatives, for use in this study and for publication of the article. The samples used in this research were not sourced from executed prisoners or prisoners of conscience.

This study was performed in compliance with relevant laws, regulatory frameworks and guidelines where the research took place. This study was approved by the Integrated Research Application System (IRAS). (Approval No. 242867)

## Declaration of competing interest

The authors declare the following financial interests/personal relationships which may be considered as potential competing interests: Christian Hedrich reports a relationship with Merck & Co Inc that includes: funding grants. CMH received unrestricted grant funding from Merck (MISP) to study lupus nephritis. If there are other authors, they declare that they have no known competing financial interests or personal relationships that could have appeared to influence the work reported in this paper.

## Data Availability

Data will be made available on request.
