# Supplementary material for: Differential transcriptional and epigenetic signatures in CD4+ T cells separate enthesitis related and psoriatic juvenile idiopathic arthritis – a pilot study
Source: J Transl Autoimmun. 2026 Aug 29;13:100397. doi: 10.1016/j.jtauto.2026.100397 (PMC13571550; doi:10.1016/j.jtauto.2026.100397)
Supplement: Supplementary file 1 [file mmc1.docx]

**Supplementary Figure 1:** **Overenrichment analysis of genes differentially expressed between patients with JIA and healthy participanys.** RNA was isolated from CD4+ nonregulatory T-cells and quantified using qubit. 50 ng of RNA was run on nanostring autoimmune arrays. Gene expression was quantified as the ratio between the gene counts of target genes and the geometric mean of housekeeping gene counts. DEGs are identified as having an adjusted p-value <0.05 and a Log2 FC >2. ORA was performed on genes identified to be increased or decreased in expression using nanostring autoimmunity panels. GO and KEGG enrichment analysis was performed of DEGs between (top) ERA and healthy participants (HCs) and (bottom) patients with jPsA and HCs.

**Supplementary Figure 2: Hierarchical clustering maps showing the top 250 differentially methylated probes between JIA patients and healthy participants.** Differentially expressed genes identified using Nanostring arrays had their DNA methylation quantified using EPIC450k arrays. Hierachical clustering of the top 250 probes between ERA and healthy participants (HC) (left) and jPsA and HC (right) (FDR < 0.05, |Δβ| > 0.05).
